# Do We Transfer Health Research Results to People?

**Published:** 2011

**Authors:** Mahnaz Ashoorkhani, Jaleh Gholami, Reza Majdzadeh

**Affiliations:** 1 Master Candidate of Health Education. Knowledge Utilization research Center, Tehran University of Medical Sciences, Tehran, Iran; 2 Candidate, Knowledge Utilization Research Center (KURC), Tehran University of Medical Sciences, Tehran, Iran; 3 Knowledge Utilization Research Center (KURC), School of Public Health, Tehran University of Medical Sciences, Tehran, Iran

## INTRODUCTION

People are very interested in health issues and medical innovations and the mass media respond likewise by offering them health news on a daily basis.^1^ The media now plays an important role in representing the health sector’s research activities publicly. The impact of the media in changing people9s way of thinking and even their behavior is undeniable. In many instances people have altered their health behavior and therapy on the grounds of news delivered by the media.^2^ Media, particularly auditory and visual influence people even in the most far flung areas.

The question remains; whether it is possible to publicly announce the results of all health research projects or not. In this study we arrived at the answer of this question through a series of in-depth interviews and focus group discussions in which health journalists and researchers9 opinions were sought.

Generally speaking, target audiences are either specific or general. Specific target audiences include peers and other researchers to which the delivery of research results is harmless; the least advantage is to prevent the repetition of research already conducted and hence save time and the heavy expenses of research. However, public media should not be used for this purpose, and they should be conveyed through specialized media such as specialized journals and academic websites. The other group of target audiences is the public. More care should be taken in announcing news to this group. The interviewees were of two different opinions in this regard. Some believe that all health research news should be conveyed to the public. Among the reasons behind this belief were creating a sense of self-belief, national identity, optimism toward the future, trust in researchers and the scientific community. However, these individuals thought there should be certain requisites to publicly announcing this type of news, such as: 1- announcing the news clearly, completely, and without exaggeration, 2- in simple and comprehensible language and without jargon terminology, 3- the public9s intellectual development and critical appraisal, 4- teaching people how to handle news published in this field.

All participant groups insisted on the method of conveying the research news. Currently, health news is not delivered properly.^3^ Participants believed that the current method was not good for specialists either.

One of the reasons stated by the participants was the 1-2 minute time limitation for announcing news in audio-visual media and the limitation of number of lines in publications. Evidently, if such limitations are considered news cannot be announced completely in detail, and consequences such as misinterpretations of the information will follow.

The other requisite in dissemination of all health news is the public9s intellectual development and critical appraisal, so that news is not valued more than what it is. People should try to confirm its authenticity. They should also be familiar with assessing the validity of research to some extent and consult experts in the field. Raising awareness to the extent that people know another study might yield different results under different circumstances. In such instances people should know that these variations are dependent on the studies and not the result of the research systems instability.

**Table 1:** Interviewee’s quotations.

“I think we should be more strict in conveying news to the public” (researcher)
“The current situation of announcing research results is not correct, even if the target audiences are specialists alone” (researcher)
“People need baked bread, not dough” (researcher)
“When people hear a research result they take it very seriously. To them, it is more than a mere news report; they begin to act upon it” (researcher)
“We should let the public know that results are prone to change and that the results may differ with a larger sample size.” (researcher)
“Research news should be conveyed properly. If and when a research process comes to a halt, the entire procedure and how and when the study has failed to accomplish should be stated, we should not be afraid of announcing a failure” (journalist)
“For example, in the case of cancer, it is wrong to announce the preliminary stages of research, even if it is a clinical trial” (journalist)
“What point is there in conveying research results to people when they are of no use to them?” (journalist)

The second group of interviewees believed that all research results should not be announced to the public. The news that is conveyed to the public should have the following characteristics; 1-it should be beneficial to the public, 2-be announced at the final and reliable stage of research, 3-be applicable to the community.

The timing of the announcement is also important. When it takes long to conduct a study people should not be notified of the preliminary results, because the results may change over time. This matter is of greater importance when it comes to therapeutic methods and drugs. The negative impact of such news is greater than the possible positive impact; over time people lose their trust in the media and research groups.

Studies that may have methodological errors or which are not applicable to the community should not be publicly published either. For example, a study conducted on a very small population is not very reliable. Also, results of studies conducted on animals should be pointed out and people should be reminded that if the same study is performed on humans it may yield different results. The media should be honest and convey all they know to the public.

On the whole, it seems that certain measures should be taken simultaneously in both domains: promoting public awareness and knowledge and encouraging critical appraisal. These measures will be useful in the long run, because the ever increasing load of information will not be easy to control. However, until that stage is reached, public health officials should control the dissemination of research results and prevent the dissemination of research that is still immature. In cases where public announcement is allowed, the results should be stated accurately, completely and without ambiguity.

## References

[1] Arnold KM (2003). Medicine in the Media: Symposium Addresses Challenge of Reporting on Medical Research. Science Editor.

[2] Haas JS, Kaplan CP, Gerstenberger EP, Kerlikowske K (2004). Changes in the Use of Postmenopausal Hormone Therapy after the Publication of Clinical Trial Results. Ann Intern Med.

[3] Robotham J, Whitehead R (2002). Media coverage of scientific presentations. MJA.

